# Supplementary material for: Proteosomal degradation of NSD2 by BRCA1 promotes leukemia cell differentiation
Source: Commun Biol. 2020 Aug 21;3:462. doi: 10.1038/s42003-020-01186-8 (PMC7443147; doi:10.1038/s42003-020-01186-8)

Supplementary Figure 1. Relation between NSD2 overexpression and leukemia maintenance

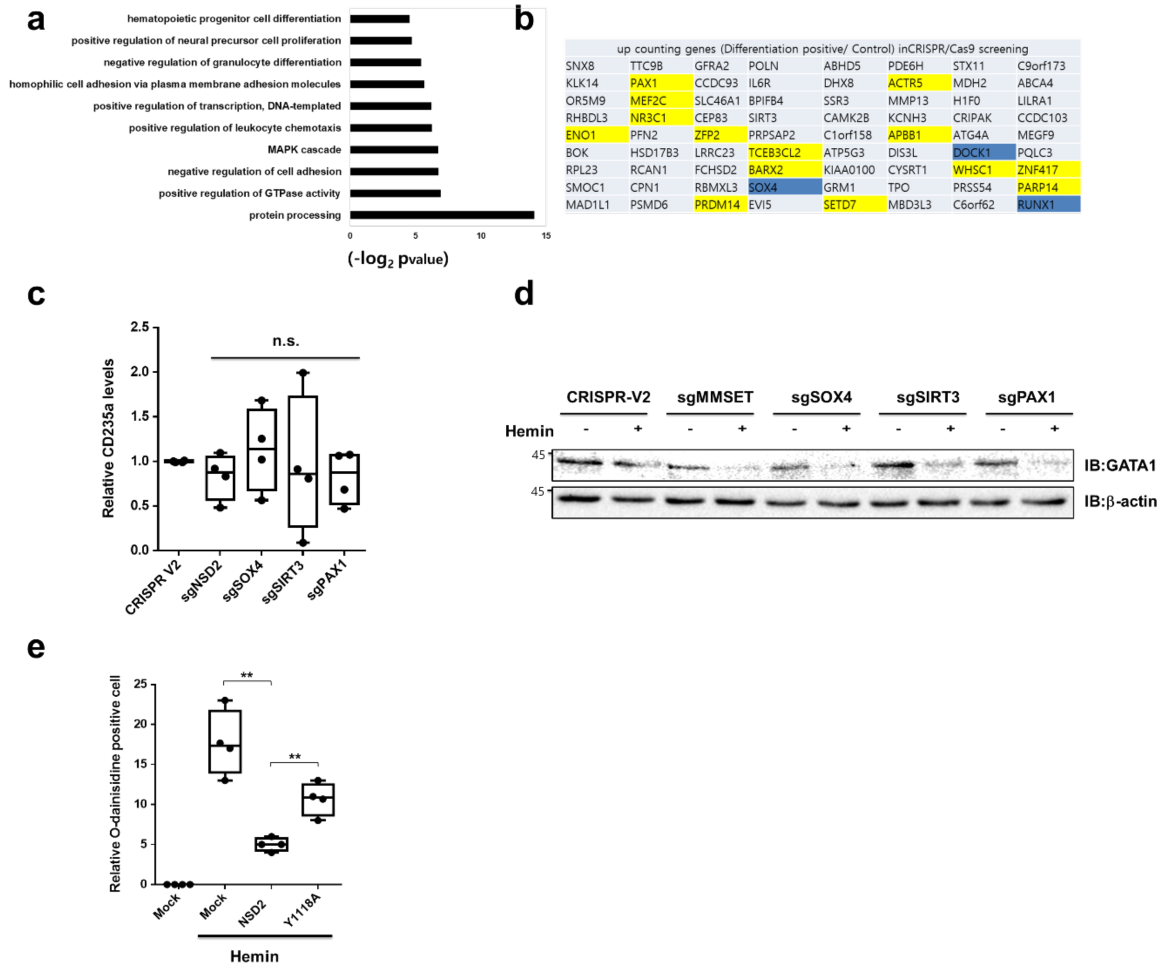

Supplementary Fig. 1. Relation between NSD2 overexpression and leukemia maintenance. **a** Gene ontology (GO) analysis depicts the significantly enriched GO terms for the top enriched and depleted genes (fold change [FC] > 2 or [FC] < 0.5) in the genome-wide CRISPR screening. Functional assignment are shown with their corresponding p values. **b** Gene lists indicate that sgRNA was significantly enriched in differentiated K562 cells. Yellow box indicates transcriptional regulators and blue box indicates erythroid differentiation related gene lists. **c** The levels of *CD235A* mRNA in K562 cells, depleting NSD2, PAX1, SIRT3, or SOX4, were quantified using real-time PCR. "n.s" means none significant. **d** GATA1 protein levels were

confirmed by immunoblotting the lysates of K562 cells, depleting NSD2, PAX1, SIRT3, or SOX4 and treated with 30 μM hemin for 2 days. **e** Cell differentiation was measured by staining K562 cells overexpressing WT or Y1118A variant of NSD2 with o-dianisidine. Quantification was shown. Results are presented as mean ± SEM, n = 3; * $p < 0.05$

Supplementary Figure 2. Effects of NSD2 in K562 differentiation

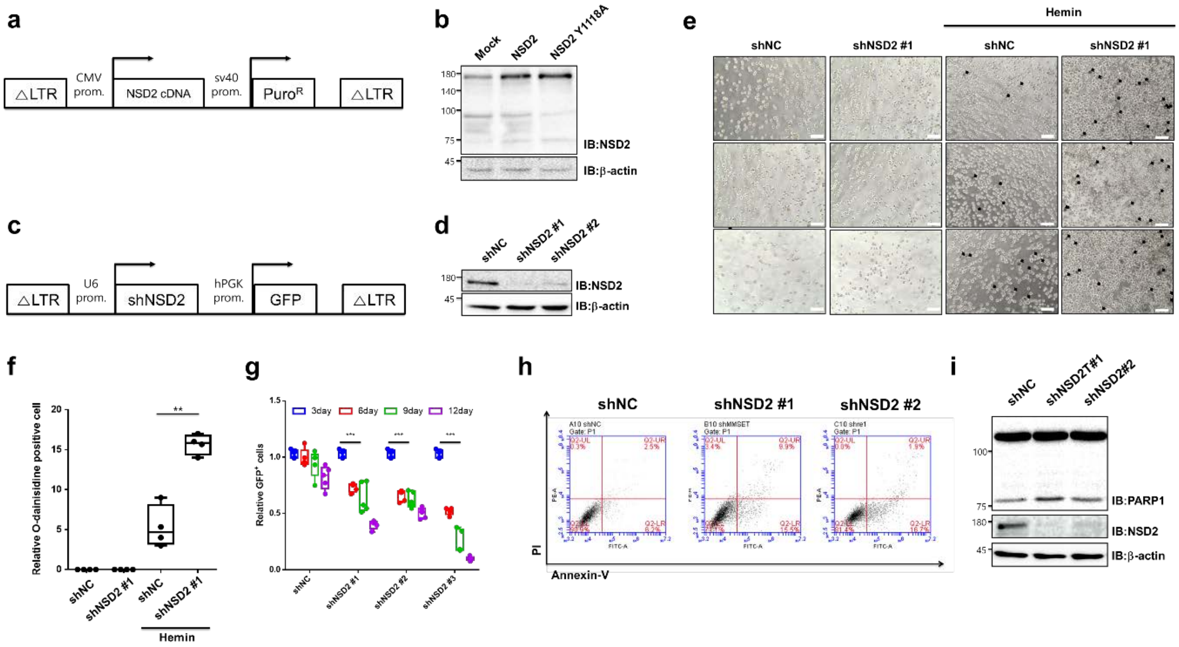

Supplementary Fig. 2. Effects of NSD2 in K562 differentiation. **a** Schematic representation of the lentiviral vector for MMSER overexpression. LTR indicates Long Terminal Repeats; Puro^{R} means resistance to puromycin. **b** NSD2 WT or Y1118A overexpressing stable K562 cells were confirmed using indicated antibodies in western blot assay. **c** Schematic representation of the lentiviral vector for NSD2 depletion. LTR indicates Long Terminal Repeats; GFP indicates Green fluorescent protein. **d** NSD2 stable knockdown K562 cells were confirmed using indicated antibodies in western blot assay. **e** O-dianisiding staining assay to NSD2 depleted K562 cells for measuring differentiation. Cells stained in brown (black arrows indicate o-dianisidine-positive cells) indicate hemoglobin accumulation. Scale bars, 25μm. **f** Quantification of o-dianisidine staining data in K562 cells. depleting NSD2 and treated with hemin. Results are presented as mean ± SEM, n = 4; ** $p <$ 0.01. **g** Cell proliferation was measured using GFP reporter vectors. GFP reporters were used in a lentiviral vector to indicate transduction of NSD2 shRNA. The ratio of GFP-positive cells was followed over time using FACS for 12 days to measure the extent of transduction of

NSD2 shRNA vector into K562 cells. The values were normalized to day 3 measurements and shNC vector was used as control. **h** Annexin-V and propidium iodide (PI) staining were quantified by FACS cytometry in NSD2 depleted K562 cells. It indicates the percentage of apoptotic cells. **i** PARP cleavage was confirmed by immunoblot in K562 cells transduced with NSD2 shRNAs. Cells were lysed and immunoblotted using indicated antibodies.

Supplementary Figure 3. K562 differentiation by NaBu (Sodium butylate) reduced NSD2 protein stability

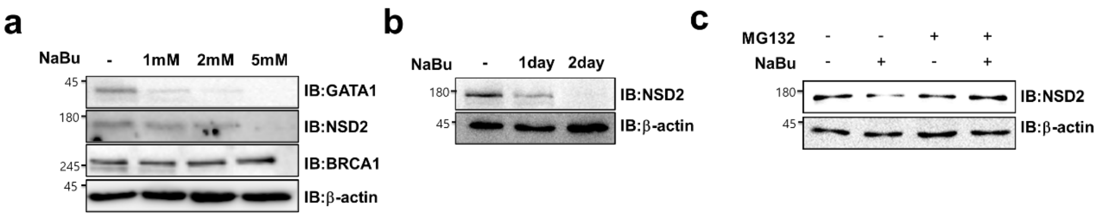

Supplementary Fig. 3. K562 differentiation by Nabu (Sodium butylate) reduced NSD2 protein stability. a Indicated protein levels were confirmed by immunoblotting the lysates of K562 cells, treated with 1 mM, 2 mM, and 5 mM Nabu for 2 days. **b** NSD2 protein levels were confirmed by immunoblotting the lysates of K562 cells, treated with 1 mM Nabu for 1 and 2 days. **c** NSD2 expression levels were confirmed by immunoblotting using indicated antibodies. The lysates of K562 cells, were treated with 1 mM Nabu for 2 days and MG132 proteasome inhibitor for 6 hr.

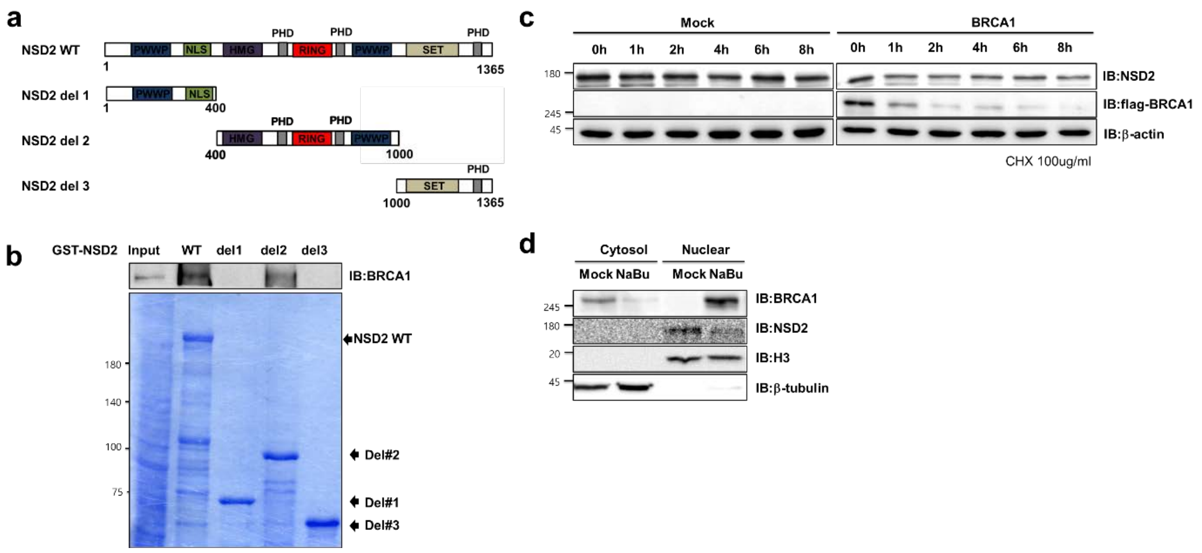

Supplementary Fig. 4. Differentiation of K562 enhances interaction with NSD2 via translocation from cytosol to nucleus. **a** Schematic representation of NSD2 or NSD2 deletion constructs. **b** Truncated GST-NSD2 variants were used for GST pull-down. Extracts of K562 cells were incubated with GST-NSD2 or GST-NSD2 deletion mutants. Associated proteins were eluted, resolved by SDS-PAGE and immunoblotted (top panel). The amount of NSD2 or NSD2 deletion mutants were determined by Coomassie staining (Bottom panel). **c** Protein stability analysis showed a reduced half-life for NSD2 in BRCA1 overexpressing 293T cells. Cell lysates were harvested after 2, 4, 6 and 8 h and analyzed by immunoblot using indicated antibodies**.** **d** Localization of NSD2 and BRCA1 was measured using Immunoblot. Total proteins from K562 cells treated with 1 mM hemin for 2 days were separated into cytoplasmic and nuclear fractions. H3 and tubulin were used as positive controls for nuclear and cytoplasmic fractions, respectively

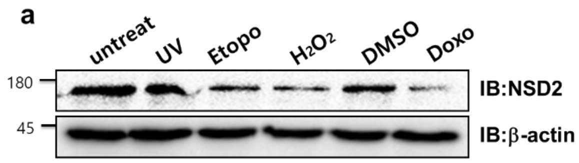

Supplementary Fig. 5. DNA damage reduces NSD2 stability. a NSD2 protein levels were confirmed by immunoblotting the lysates of K562 cells, treated with 100 J/m^2 UV, 10 uM Etoposide, 100 uM H2O2 or 1nM Doxorubicin for 1day.

Supplementary Figure 6. NSD2 indirectly regulates erythrocyte differentiation-related gene expression through regulation of AURKA.

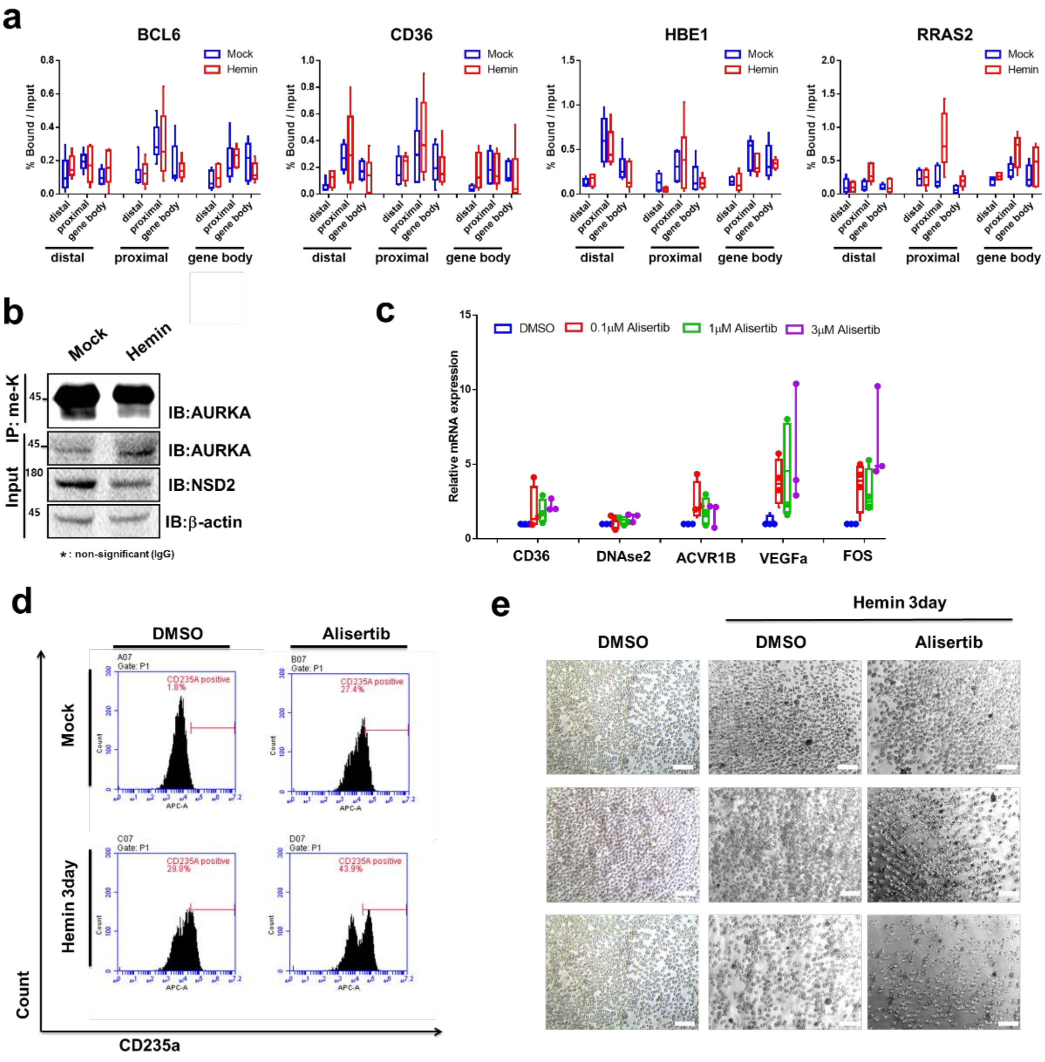

Supplementary Fig. 6. NSD2 indirectly regulates erythrocyte differentiation-related gene expression through regulation of AURKA. **a** K562 cells were treated with 30 μM hemin. ChIP analysis employing anti-IgG, anti-NSD2, and anti-H3K36me2 antibodies was performed. The immunoprecipitated DNA fragments from the two promoters and gene-body regions of BCL6, CD36, HBE1 and RRAS2 were analyzed by real-time PCR. Values represent mean ± SD of technical triplicates from a representative experiment. All experiments were performed three times with similar results. **b** Immunoprecipitation analysis showed methylation of AURKA.. K562 cells treated with 30 μM hemin and 10 μM MG132 were immunoblotted with anti-meK antibody. **c** K562 cells were treated with indicated concentration of alisertib for 2

days. The mRNA levels of differentiation-related genes were analyzed by real-time PCR and normalized to those of β-actin. **d** Induction of cell differentiation by alisertib was measured by staining K562 cells treated with hemin and alisertib with anti-CD235A for 1 hr and sorting by FACS. **e** O-dianisiding staining assay to K562 cells treated with alisertib and hemin for measuring differentiation. Cells stained in brown indicate hemoglobin accumulation. Scale bars, 25μm.

Supplementary Figure7. BRCA1 was overexpressed or mutated in hematopoietic and lymphoid cancers

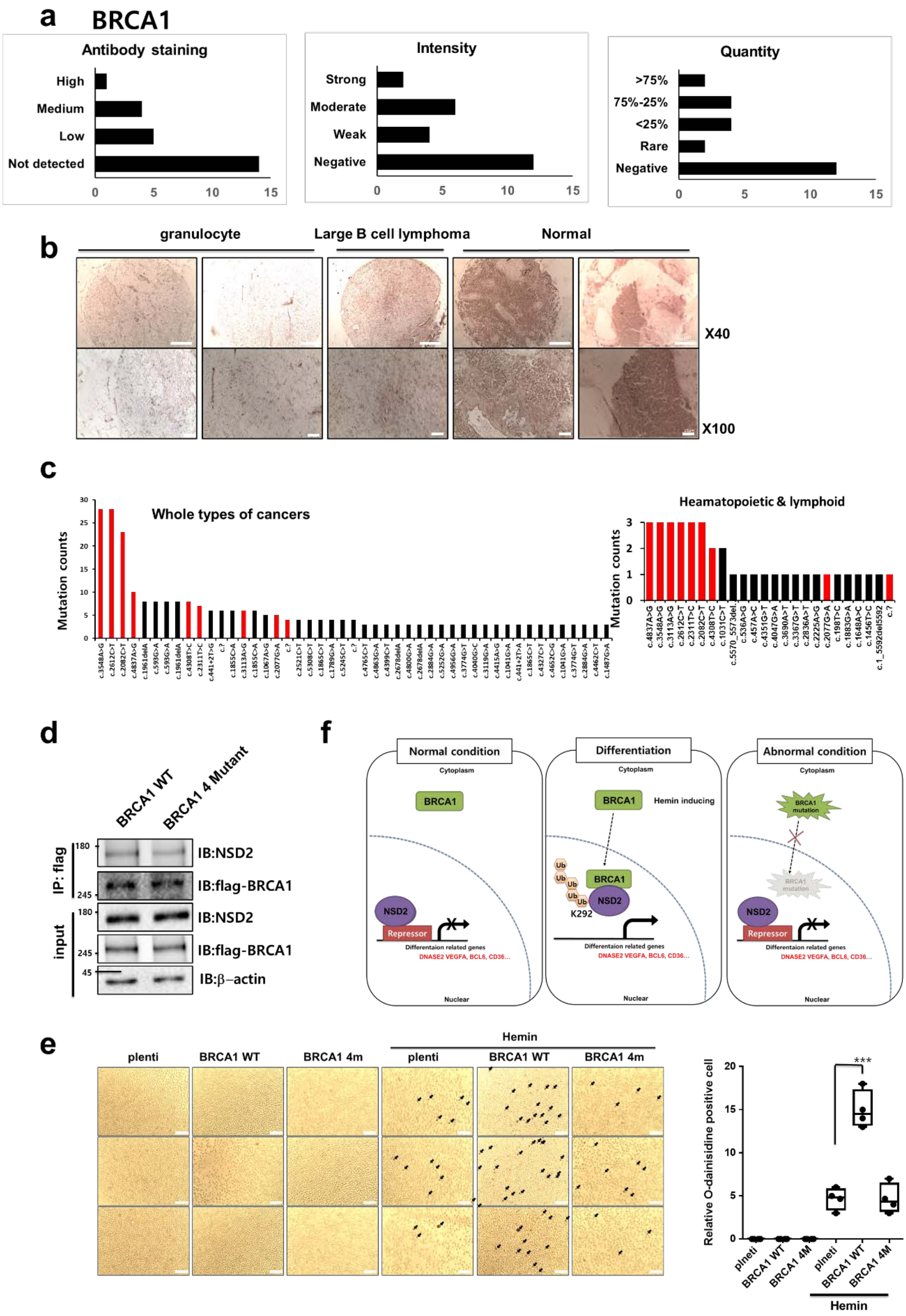

99

Supplementary Fig. 7. BRCA1 is overexpressed or mutated in hematopoietic and lymphoid cancers. **a** BRCA1 expression levels according to the Human Protein Atlas database are shown. Protein expression score is based on the immunohistochemical data manually scored with regard to staining intensity (negative, weak, moderate, or strong) and fraction of stained cells (< 25%, 25–75% or > 75%). Each combination of intensity and stained fraction is automatically converted into a protein expression level score as follows: negative not detected; weak < 25% - not detected; weak combined with either 25–75% or 75% - low; moderate < 25% - low; moderate combined with either 25–75% or 75% - medium; strong < 25% - medium; strong combined with either 25–75% or 75% - high. **b** Formalin-fixed tissue array slides from granulocyte and large B cell lymphoma and normal tissues were used for 3,3-diaminobenzidine (DAB) staining of BRCA1 for immune-histochemical analysis. **c** TCGA database provided an overview of genomic alterations in whole type of cancer (left) and hematopoietic and lymphoid cancer (right). **d** Interaction between of NSD2 and BRCA1 or 4 different mutants was confirmed using immunoprecipitation assay . The 293T cells were treated with 10 μM MG132 for 3 hr before harvest. **e** Cell differentiation was measured by staining K562 overexpressing WT or mutants BRCA1 or control cells, with o-dianisidine. Cells stained in brown (o-dianisidine-positive cells) indicate hemoglobin accumulation (left). Quantification data was shown (right). Scale bars, 25μm. **f** Schematic representation for K562 cell differentiation mechanism that hemin-mediated BRCA1 translocation into cytosol, followed by inducing NSD2 polyubiquitination.

Name	Sequencing (5’ to 3’)	purpose
**shMMSET #1 sense**	CCGGGGGCATTGTTCAAGCAGAAGACTCGAGCTTCTGCTTGAACAATGCCCTTTTTG	**shRNA**
**shMMSET #1 anti-sense**	AATTCAAAAAGGGCATTGTTCAAGCAGAAGACTCGAGTCTTCTGCTTGAACAATGCCC	**shRNA**
**shMMSET #2 sense**	CCGGCGGAAAGCCAAGTTCACCTTTCTCGAGAAAGGTGAACTTGGCTTTCCGTTTTTG	**shRNA**
**shMMSET #2 anti-sense**	AATTCAAAAACGGAAAGCCAAGTTCACCTTTCTCGGAAAGGTGAACTTGGCTTTCCG	**shRNA**
**shBRCA1 CDS F**	CCGG GAGTATGCAAACAGCTATAAT CTCGAG ATTATAGCTGTTTGCATACTC TTTTTG	**shRNA**
**shBRCA1 CDS R**	AATTCAAAAA GAGTATGCAAACAGCTATAAT CTCGAG ATTATAGCTGTTTGCATACTC	**shRNA**
**shBRCA1 UTR F**	CCGG CCCTAAGTTTACTTCTCTAAA CTCGAG TTTAGAGAAGTAAACTTAGGG TTTTTG	**shRNA**
**shBRCA1 UTR R**	AATTCAAAAA CCCTAAGTTTACTTCTCTAAA CTCGAG TTTAGAGAAGTAAACTTAGGG	**shRNA**
**BCL6 proximal Promoter F**	AGGGACCTGAGTTGCATTCTG	**ChIP**
**BCL6 proximal Promoter R**	GAACCTGTTTCCTGCTTGCC	**ChIP**
**BCL6 distal Promoter F**	CTGAAGAGCCACCTGCGAAT	**ChIP**
**BCL6 distal Promoter R**	CATTAGCGTAGTGGTTGCCC	**ChIP**
**BCL6 genebody F**	CTGGAGATGGTATTGCCGCC	**ChIP**
**BCL6 genebody R**	TGTGGGAAGGGCTACGAATC	**ChIP**
**CD36 proximal Promoter F**	GCCAGTCTTGAGGTCCTACAT	**ChIP**
**CD36 proximal Promoter R**	GAGTGCATCAACTACAAAGACAT	**ChIP**
**CD36 distal Promoter F**	ACTGAATGGATACCTTGCCC	**ChIP**
**CD36 distal Promoter R**	GCTCTGCCAACTCAAGAAGT	**ChIP**
**CD36 genebody F**	AGGTGAGTGAGTCCCCACAA	**ChIP**
**CD36 genebody R**	TGACATTTGCCAAGTAGAAGACT	**ChIP**
**HBE1 proximal Promoter F**	AGAGGATTCTCTGGAAGCACTG	**ChIP**
**HBE1 proximal Promoter R**	TCCACAGTGGGACTAAAGCC	**ChIP**
**HBE1 distal Promoter F**	GATGGGCTAGAGTTCCTCTTTC	**ChIP**
**HBE1 distal Promoter R**	GTCACTGGGCAATACAAGACCT	**ChIP**
**HBE1 genebody F**	GGGTGAGGGTGTAGGTAGGT	**ChIP**
**HBE1 genebody R**	GGTTTGTGCCCACCCAAAAA	**ChIP**
**RRAS2 proximal Promoter F**	AGTGGGTGTCAGTTGGGAGT	**ChIP**
**RRAS2 proximal Promoter R**	CCACACAATCCCTTACATAGACAA	**ChIP**
**RRAS2 distal Promoter F**	ATATAGGCAATCTCTCAGTCCCT	**ChIP**
**RRAS2 distal Promoter R**	CCCTGGAGAGAGTGTGGAAC	**ChIP**
**RRAS2 genebody F**	ATAGATGACAGAGCAGCCCG	**ChIP**
**RRAS2 genebody R**	ACATGGGCTAATATTCCAGATCA	**ChIP**
**VEGFA F**	ACGAAAGCGCAAGAAATCCC	**RT-PCR**
**VRGFA R**	GGAGGCTCCAGGGCATTAG	**RT-PCR**
**DNAse2 F**	AGCCAAGAACCCTGGAACAG	**RT-PCR**
**DNAse2 R**	GCCGGAGTACAGGTCATCTC	**RT-PCR**
**FOS F**	CTTTGCAGACCGAGATTGCC	**RT-PCR**
**FOS R**	ATCAGGGATCTTGCAGGCAG	**RT-PCR**
**BCL6 F**	ATGAGGAGTTTCGGGATGTC	**RT-PCR**
**BCL6 R**	TGCCTCTTCTGGGATTGTTT	**RT-PCR**
**PIM F**	GCTGTGCTGGGAGAAATACT	**RT-PCR**
**PIM R**	GGTCTTGGCTTTGAAACAGT	**RT-PCR**
**CD36 F**	TGTCCTGGCTGTGTTTGGAG	**RT-PCR**
**CD36 R**	AGACTGTGTTGTCCTCAGCG	**RT-PCR**
**P5 0 nt stagger**	AATGATACGGCGACCACCGAGATCTACACTCTTTCCCTACACGACGCTCTTCCGATCTTTGTG GAAAGGACGAAACACCG	**illumina sequencing**
**P5 1nt stagger**	AATGATACGGCGACCACCGAGATCTACACTCTTTCCCTACACGACGCTCTTCCGATCTCTTGT GGAAAGGACGAAACACCG	**illumina sequencing**
**P5 2 nt stagger**	AATGATACGGCGACCACCGAGATCTACACTCTTTCCCTACACGACGCTCTTCCGATCTGCTT GTGGAAAGGACGAAACACCG	**illumina sequencing**
**P5 3 nt stagger**	AATGATACGGCGACCACCGAGATCTACACTCTTTCCCTACACGACGCTCTTCCGATCTAGCTT GTGGAAAGGACGAAACACCG	**illumina sequencing**
**P5 4 nt stagger**	AATGATACGGCGACCACCGAGATCTACACTCTTTCCCTACACGACGCTCTTCCGATCTCAAC TTGTGGAAAGGACGAAACACCG	**illumina sequencing**
**P5 6 nt stagger**	AATGATACGGCGACCACCGAGATCTACACTCTTTCCCTACACGACGCTCTTCCGATCTTGCA CCTTGTGGAAAGGACGAAACACCG	**illumina sequencing**
**P5 7 nt stagger**	AATGATACGGCGACCACCGAGATCTACACTCTTTCCCTACACGACGCTCTTCCGAT CTACG	**illumina sequencing**
**P5 8 nt stagger**	AATGATACGGCGACCACCGAGATCTACACTCTTTCCCTACACGACGCTCTTCCGAT CTACG	**illumina sequencing**
**P7 primer**	CAAGCAGAAGACGGCATACGAGATNNNNNNNNGTGACTGGAGTTC AGACGTGTGCTCTTCCGATCTTCTACTATTCTTTCCCCTGCACTGT	**illumina sequencing**
**C01**	GCACGACC	**Barcod sequence**
**D01**	TATGGACC	**Barcod sequence**

Supplementary Table 1. Primer sequence was used this article.

Accession	Entry	Description	Peptides	Coverage (%)
H0Y599	H0Y599	Dedicator of cytokinesis protein 4 Fragment OS Homo sapiens GN DOCK4 PE 4 SV 1	56	22.2611
Q15643	TRIPB	Thyroid receptor interacting protein 11 OS Homo sapiens GN TRIP11 PE 1 SV 3	41	20.0101
P0CG48	UBC	Polyubiquitin C OS Homo sapiens GN UBC PE 1 SV 3	41	50.8029
C9JX92	C9JX92	Afadin OS Homo sapiens GN MLLT4 PE 2 SV 1	37	15.4332
P38398	BRCA1	Breast cancer type 1 susceptibility protein OS Homo sapiens GN BRCA1 PE 1 SV 2	36	16.2104
O96028	NSD2	Histone lysine N methyltransferase NSD2 OS Homo sapiens GN WHSC1 PE 1 SV 1	32	18.9011
Q7Z6E9	RBBP6	E3 ubiquitin protein ligase RBBP6 OS Homo sapiens GN RBBP6 PE 1 SV 1	30	11.3281
Q5VTR2	BRE1A	E3 ubiquitin protein ligase BRE1A OS Homo sapiens GN RNF20 PE 1 SV 2	28	22.8718
Q14139	UBE4A	Ubiquitin conjugation factor E4 A OS Homo sapiens GN UBE4A PE 1 SV 2	26	19.8874
P0CG48	UBC	Polyubiquitin C OS Homo sapiens GN UBC PE 1 SV 3	24	40.292
O95155	UBE4B	Ubiquitin conjugation factor E4 B OS Homo sapiens GN UBE4B PE 1 SV 1	22	17.0507
P0CG48	UBC	Polyubiquitin C OS Homo sapiens GN UBC PE 1 SV 3	21	31.3869
Q86W50	MET16	Methyltransferase like protein 16 OS Homo sapiens GN METTL16 PE 1 SV 2	19	21.5303
P10412	H14	Histone H1 4 OS Homo sapiens GN HIST1H1E PE 1 SV 2	17	22.831
A3KFJ0	A3KFJ0	Aurora kinase A OS Homo sapiens GN AURKA PE 2 SV 1	15	30.2594
Q9HC52	CBX8	Chromobox protein homolog 8 OS Homo sapiens GN CBX8 PE 1 SV 3	15	34.1902
H3BT71	RBMX	RNA binding motif protein X chromosome N terminally processed OS Homo sapiens GN RBMX PE 2 SV 1	13	25
B4DR52	B4DR52	Histone H2B OS Homo sapiens GN HIST2H2BF PE 2 SV 1	12	40.3614
J3QQJ0	J3QQJ0	SAP30 binding protein Fragment OS Homo sapiens GN SAP30BP PE 4 SV 1	11	31.0769
P16104	H2AX	Histone H2A x OS Homo sapiens GN H2AFX PE 1 SV 2	11	34.965
Q9HC52	CBX8	Chromobox protein homolog 8 OS Homo sapiens GN CBX8 PE 1 SV 3	7	20.0514
H0YLV8	BLM	Bloom syndrome protein OS Homo sapiens GN BLM PE 2 SV 1	7	10.8959
Q96T66	NMNA3	Nicotinamide mononucleotide adenylyltransferase 3 OS Homo sapiens GN NMNAT3 PE 1 SV 2	5	20.6349
Q8NFK1	CXG3	Gap junction gamma 3 protein OS Homo sapiens GN GJC3 PE 2 SV 1	3	6.4516

Supplementary Table 2. MMSET interaction partner lists was found in LC-MS/MS

Data for Figure 2

Figure 2A

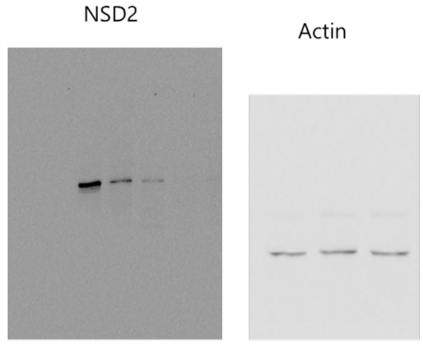

Figure 2B

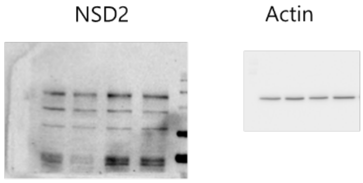

Figure 2C

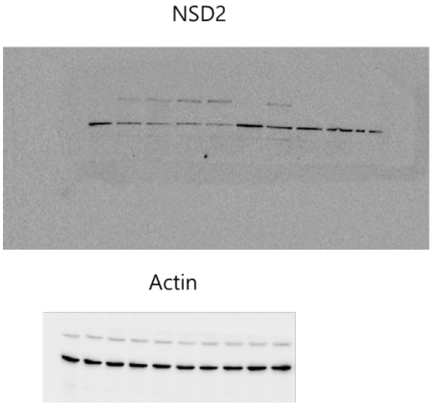

Figure 2D

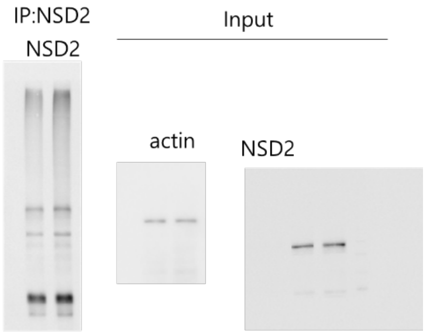

Data for Figure 3

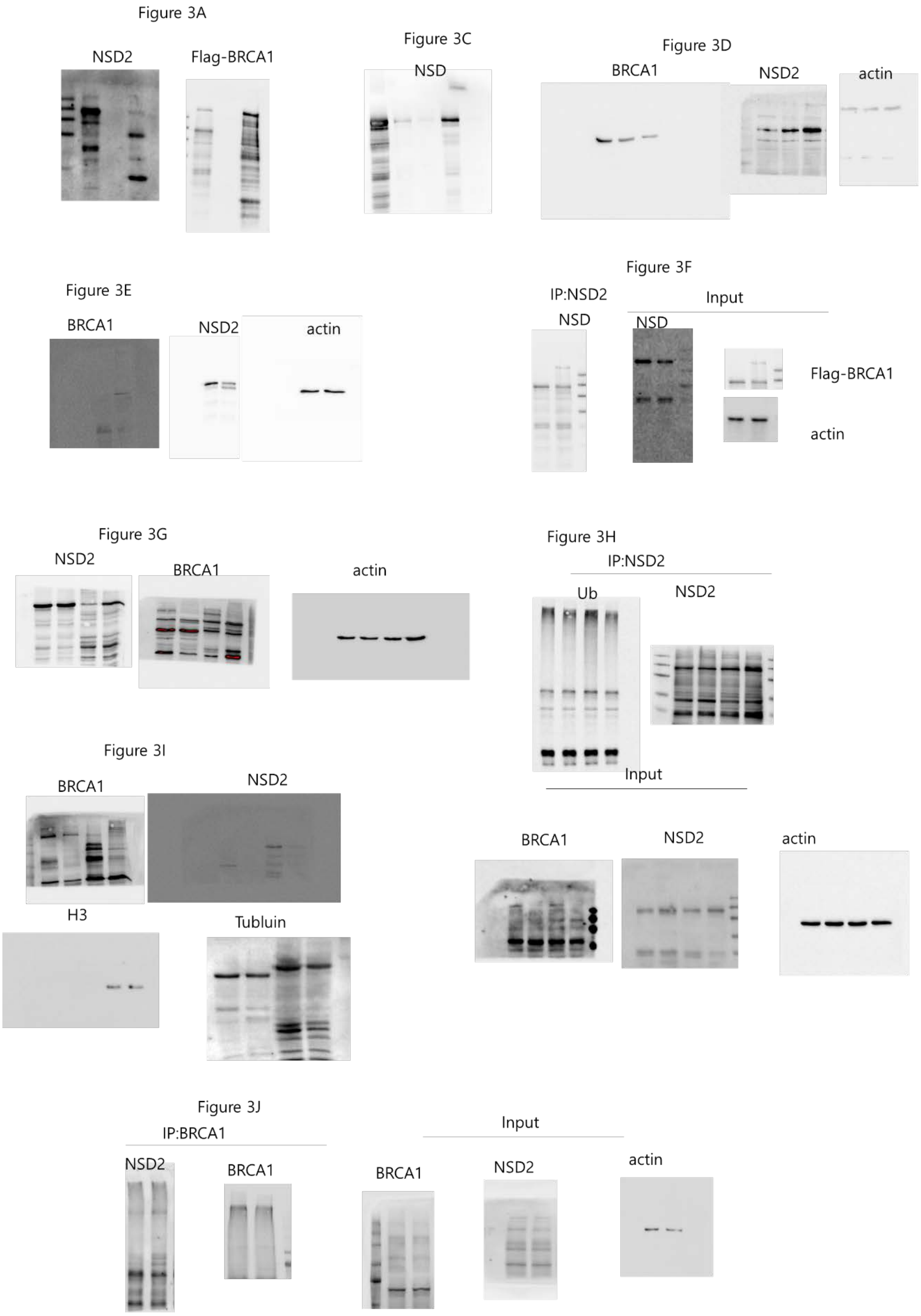

Data for Figure 4

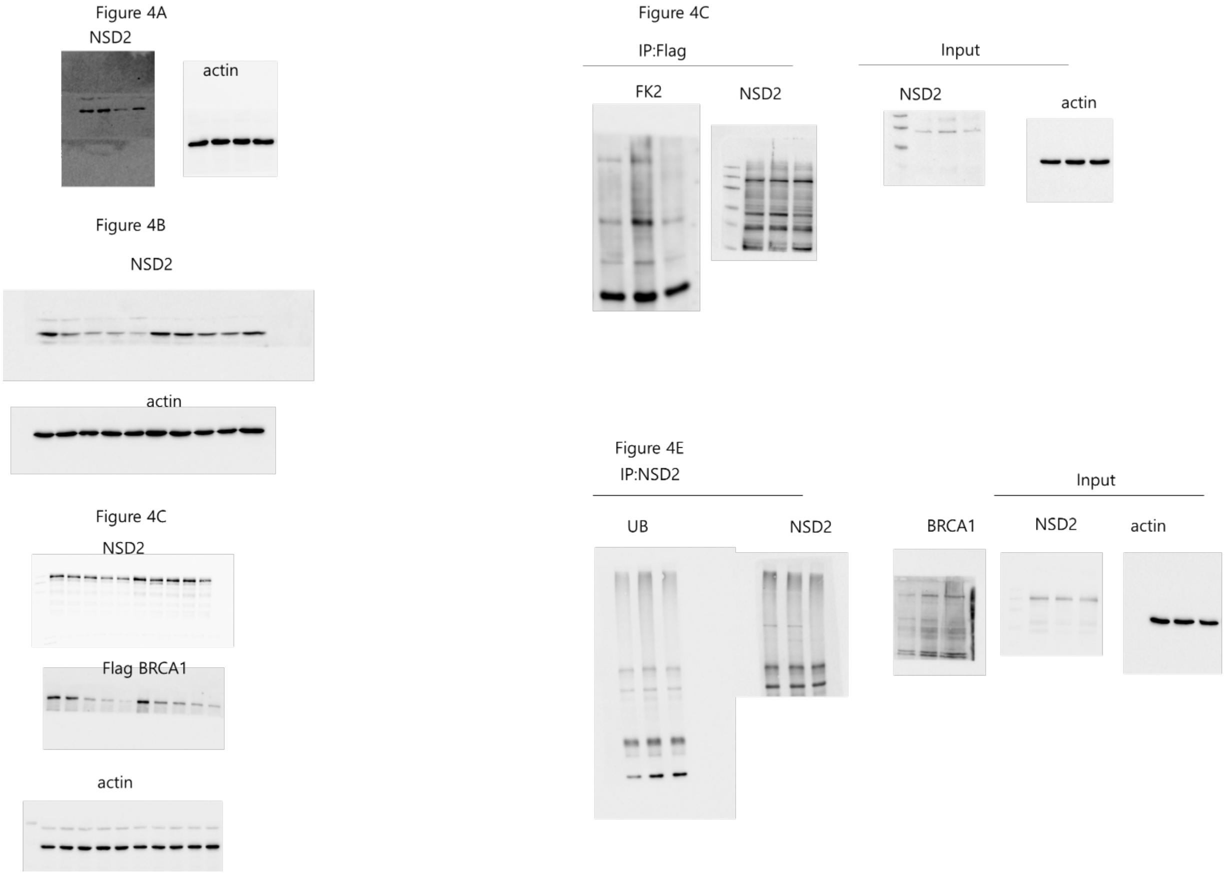

Data for Figure 6

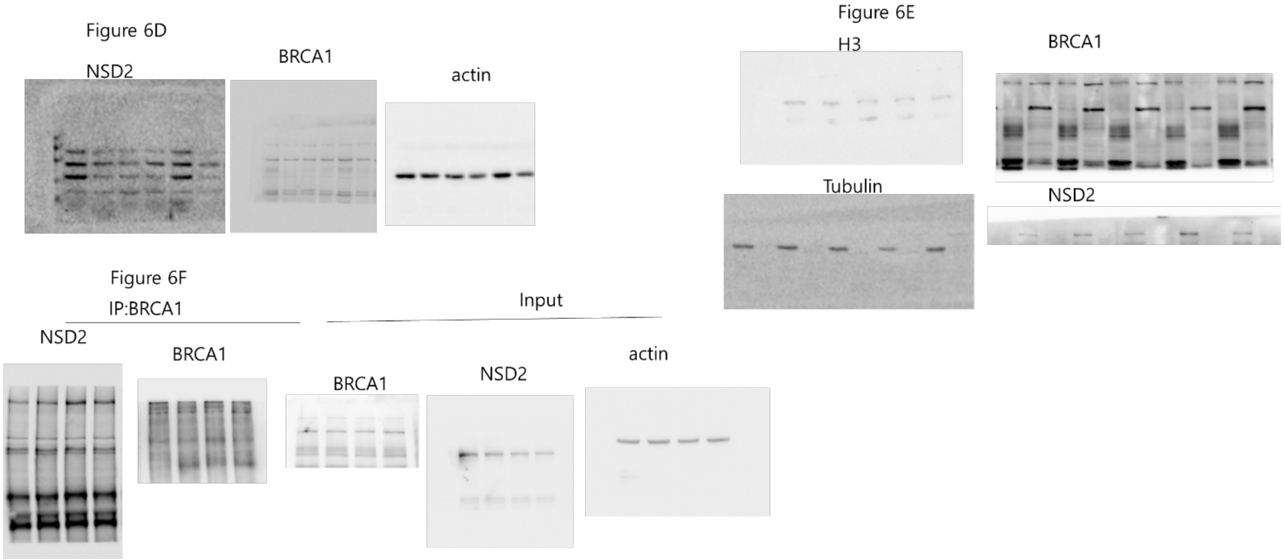

Supplement: Supplementary file 1 — Supplementary Information [file 42003_2020_1186_MOESM1_ESM.pdf]
