# Supplementary material for: Proteosomal degradation of NSD2 by BRCA1 promotes leukemia cell differentiation
Source: Commun Biol. 2020 Aug 21;3:462. doi: 10.1038/s42003-020-01186-8 (PMC7443147; doi:10.1038/s42003-020-01186-8)
Supplement: Supplementary file 2 — Description of Additional Supplementary Files [file 42003_2020_1186_MOESM2_ESM.docx]

**Description of Additional Supplementary Files**

**File Name: Supplementary Data 1**

**Description:** Raw Data underlying the graphs.

**File Name: Supplementary Data 2**

**Description**: CRISPR sgRNA sequences
