# Supplementary material for: Proteosomal degradation of NSD2 by BRCA1 promotes leukemia cell differentiation
Source: Commun Biol. 2020 Aug 21;3:462. doi: 10.1038/s42003-020-01186-8 (PMC7443147; doi:10.1038/s42003-020-01186-8)
Supplement: Supplementary file 5 — Reporting Summary [file 42003_2020_1186_MOESM5_ESM.pdf]

nature research

Corresponding author(s): Sang-Beom Seo COMMSBIO-19-1384B

Last updated by author(s): Jun 20, 2020

Reporting Summary

Nature Research wishes to improve the reproducibility of the work that we publish. This form provides structure for consistency and transparency in reporting. For further information on Nature Research policies, see Authors & Referees and the Editorial Policy Checklist .

Statistics

For all statistical analyses, confirm that the following items are present in the figure legend, table legend, main text, or Methods section.

n/a	Confirmed	
☐	☒	The exact sample size (n) for each experimental group/condition, given as a discrete number and unit of measurement
☐	☒	A statement on whether measurements were taken from distinct samples or whether the same sample was measured repeatedly
☐	☒	The statistical test(s) used AND whether they are one- or two-sided<br>*Only common tests should be described solely by name; describe more complex techniques in the Methods section.*
☐	☒	A description of all covariates tested
☐	☒	A description of any assumptions or corrections, such as tests of normality and adjustment for multiple comparisons
☐	☒	A full description of the statistical parameters including central tendency (e.g. means) or other basic estimates (e.g. regression coefficient) AND variation (e.g. standard deviation) or associated estimates of uncertainty (e.g. confidence intervals)
☐	☒	For null hypothesis testing, the test statistic (e.g. F, t, r) with confidence intervals, effect sizes, degrees of freedom and P value noted<br>*Give P values as exact values whenever suitable.*
☒	☐	For Bayesian analysis, information on the choice of priors and Markov chain Monte Carlo settings
☒	☐	For hierarchical and complex designs, identification of the appropriate level for tests and full reporting of outcomes
☒	☐	Estimates of effect sizes (e.g. Cohen's d, Pearson's r), indicating how they were calculated

Our web collection on statistics for biologists contains articles on many of the points above.

Software and code

Policy information about availability of computer code

Data collection	GEO data (GSE92878, GSE144939), Human protein altas (HPA057371, CAB001946), COSMIC data for identifying BRCA1 mutation in cancer patients
Data analysis	We used EXCEL for combining data set

For manuscripts utilizing custom algorithms or software that are central to the research but not yet described in published literature, software must be made available to editors/reviewers. We strongly encourage code deposition in a community repository (e.g. GitHub). See the Nature Research guidelines for submitting code & software for further information.

Data

Policy information about availability of data

All manuscripts must include a data availability statement. This statement should provide the following information, where applicable:

- Accession codes, unique identifiers, or web links for publicly available datasets
- A list of figures that have associated raw data
- A description of any restrictions on data availability

NGS sequencing data have been deposited in Supplementary Data 2. Microarray data have been deposited in xxx with accession number GSE92878 and GSE133939. Source data can be found in Supplementary Data 1. All other data are available within the manuscript files or from the corresponding author upon reasonable request.

Field-specific reporting

Please select the one below that is the best fit for your research. If you are not sure, read the appropriate sections before making your selection.

- [x] Life sciences
- [ ] Behavioural & social sciences
- [ ] Ecological, evolutionary & environmental sciences

For a reference copy of the document with all sections, see nature.com/documents/nr-reporting-summary-flat.pdf

Life sciences study design

All studies must disclose on these points even when the disclosure is negative.

Sample size	We repeated 3 to 6 experiments and used t-test to verify the accuracy of the experiments.
Data exclusions	We used all the data
Replication	We usually performed three independent experiments and got similar results. At the same time, a positive experiment was performed to confirm the success of the experiment
Randomization	We conducted experiments using K562 and 293T cells. Even in the case of Knockdown stable cells, we made several time for independent expriments.
Blinding	Several authors confirmed the experimental data through cross-validation.

Reporting for specific materials, systems and methods

We require information from authors about some types of materials, experimental systems and methods used in many studies. Here, indicate whether each material, system or method listed is relevant to your study. If you are not sure if a list item applies to your research, read the appropriate section before selecting a response.

Materials & experimental systems

n/a	Involved in the study
☐	☒ Antibodies
☐	☒ Eukaryotic cell lines
☒	☐ Palaeontology
☒	☐ Animals and other organisms
☒	☐ Human research participants
☒	☐ Clinical data

Methods

n/a	Involved in the study
☒	☐ ChIP-seq
☐	☒ Flow cytometry
☒	☐ MRI-based neuroimaging

Antibodies

Antibodies used	Antibodies against H3K36me2 (Millipore, Billerica, MA; 07-274), BRCA1 (07-434), Flag (Sigma, St. Louis, MO; F3165), MMSET (EpiCypher, Durham, NC; 13-0002), MMSET/NDS2 (Abcam, ab75359), APC-CD235A (eBioscience, Waltham, MA;17-9987-42), FK2 (Enzo life sciences, Farmingdale, NY; BML-pw-8810-0100,), b-actin (sc-47778), H3 (sc-8654), PARP1 (sc-56197), and tubulin (sc-9103) (all from Santa Cruz Biotechnology, Dallas, TX) were employed.
Validation	We used all antibodies by referring to manufacturer's website

Eukaryotic cell lines

Policy information about cell lines

Cell line source(s)	K562, HEK293T
Authentication	K562 and HEK293T was purchased in Korea cell line bank (KCLB NO. 10243, 21573)
Mycoplasma contamination	We are treating ciprofloxacin for removing mycoplasma and checking regularly mycoplasma contamination
Commonly misidentified lines (See ICLAC register)	*Name any commonly misidentified cell lines used in the study and provide a rationale for their use.*

Flow Cytometry

Plots

Confirm that:

- [x] The axis labels state the marker and fluorochrome used (e.g. CD4-FITC).
- [x] The axis scales are clearly visible. Include numbers along axes only for bottom left plot of group (a 'group' is an analysis of identical markers).
- [ ] All plots are contour plots with outliers or pseudocolor plots.
- [x] A numerical value for number of cells or percentage (with statistics) is provided.

Methodology

Sample preparation

To CRISPR-Cas9 screening, K562 cells were infected with lenti-CRISPR-V2 virus and selected using 1 µg/mL puromycin for making Cas9-expressing K562 cells. Cas9-expressing K562 cells were infected with sgRNA library virus (at a multiplicity of infection of 0.3) using polybrene at a density equivalent to 2.5 X 107 cells (~300 cells per a sgRNA) per 150 mm plate area. After 3 days, we cultured the K562 cells of 2.5 X 107 per each 150 mm plate and treated with NaOH or hemin for 3days. For differentiated cell sorting, hemin-treated cells were washed with PBS, resuspended cells in 1X binding buffer, stained with CD235A-APC Abs for 30 min at RT in the dark.
The CD235A+ cells were separated on FACS Aria III (BD biosciences) and positive APC staining (total CD235A+ cells). Purity of sorted fractions as verified by FACS analysis was more than 50%.
To measure ratio of K562 differentiation, K562 cells were induced by MMSET WT, Y1118A or K292R and treated with 30 mM hemin. The cells were washed with PBS, resuspend cells in 1X binding buffer, added APC-CD235A for 30min at RT in the dark. The cells were subjected to FACS analysis using a FACSCalibur system.
To measure the effect of MMSET on apoptosis, K562 shNC and shMMSET cells were washed with PBS, resuspend cells in 1X binding buffer, added FITC-Anenexin V and PI (BD Bioscience) for 30min at RT in the dark. The cells were subjected to FACS analysis using a FACSCalibur system.

Instrument

FACS Aria III (BD biosciences) is used for cell sorting, BD Accuri C6 plus is used for measuring apoptosis and differentiation

Software

we used BD Accuri C6 plus software which was offered by manufacturer

Cell population abundance

10,000 cells were used for cell analysis such as apoptosis and K562 diferentiation. and sgRNA library has 76,441 gRNAs. To achieve the recommended representation of -300 cell prer sgRNA, total 2.5X10^7 cells were used for cell sorting

Gating strategy

We gated to measure the living cell using FSC, SSC. additionally, we gated FSC-H, FSC-A for identifying single cell.
For measure cell apoptosis, we tried Annexin-V or PI or Annexing-V and PI double staining. Each single staining value was used in fluorescence compensation.
To measure K562 differentiation, we used APC-CD235A antibody. we compare the values with unstained samples.

- [ ] Tick this box to confirm that a figure exemplifying the gating strategy is provided in the Supplementary Information.
